# Detection of breast cancer in digital breast tomosynthesis with vision transformers

**DOI:** 10.1038/s41598-024-72707-2

**Published:** 2024-09-27

**Authors:** Idan Kassis, Dror Lederman, Gal Ben-Arie, Maia Giladi Rosenthal, Ilan Shelef, Yaniv Zigel

**Affiliations:** 1 Department of Biomedical Engineering, Ben Gurion University of the Negev, Be’er-Sheva, 8410501 Israel; 2 Faculty of Engineering, Holon Institute of Technology, Holon, 5810201 Israel; 3 Imaging Institute, Soroka Medical Center, Be’er-Sheva, 84101 Israel; 4 Breast Health Center, Soroka Medical Center, Be’er-Sheva, 84101 Israel

**Keywords:** Cancer imaging, Biomedical engineering, Information technology

## Abstract

Digital Breast Tomosynthesis (DBT) has revolutionized more traditional breast imaging through its three-dimensional (3D) visualization capability that significantly enhances lesion discernibility, reduces tissue overlap, and improves diagnostic precision as compared to conventional two-dimensional (2D) mammography. In this study, we propose an advanced Computer-Aided Detection (CAD) system that harnesses the power of vision transformers to augment DBT’s diagnostic efficiency. This scheme uses a neural network to glean attributes from the 2D slices of DBT followed by post-processing that considers features from neighboring slices to categorize the entire 3D scan. By leveraging a transfer learning technique, we trained and validated our CAD framework on a unique dataset consisting of 3,831 DBT scans and subsequently tested it on 685 scans. Of the architectures tested, the Swin Transformer outperformed the ResNet101 and vanilla Vision Transformer. It achieved an impressive AUC score of 0.934 ± 0.026 at a resolution of 384 $\times$ 384. Increasing the image resolution from 224 to 384 not only maintained vital image attributes but also led to a marked improvement in performance (p-value = 0.0003). The Mean Teacher algorithm, a semi-supervised method using both labeled and unlabeled DBT slices, showed no significant improvement over the supervised approach. Comprehensive analyses across different lesion types, sizes, and patient ages revealed consistent performance. The integration of attention mechanisms yielded a visual narrative of the model’s decision-making process that highlighted the prioritized regions during assessments. These findings should significantly propel the methodologies employed in DBT image analysis by setting a new benchmark for breast cancer diagnostic precision.

## Introduction

Breast cancer ranks as the most prevalent malignancy among women worldwide and imposes a substantial burden on the overall health of the population^1,2^. About one in eight women will be diagnosed with breast cancer at some point. Early diagnosis is a crucial, pivotal strategy in lessening breast cancer’s overall disease burden and mortality rates, and can prevent deaths through effective detection and state-of-the-art cancer treatment^3,4^. Therefore, the preventive behaviors and screening programs implemented worldwide are essential to curbing breast cancer incidence rates and initiating early treatment^5^.

Mammography, which is currently the predominant screening and diagnostic tool, entails using low-dose X-rays to visualize the breast tissues. It is the prime noninvasive, instrumental method for the early detection of numerous breast cancers^6^. However, the two-dimensional nature of mammography, as compared to the three-dimensional structure of the breast, can make tumor detection difficult, particularly for dense breasts^7,8^. DBT is an emerging technique that addresses the limitations caused by overlapping structures in mammography^9^. In DBT, multiple projection views are acquired as the X-ray source moves along a predefined trajectory that typically spans an angular range of 60°. The obtained projection views are reconstructed to provide sections that parallel the breast . Overall, DBT has shown promise in improving the diagnostic accuracy of breast cancer detection in specific subgroups of women, including those with dense breasts^10^. During standard screening, mammograms and tomosynthesis are typically obtained in the Craniocaudal (CC) and Medial-lateral-oblique (MLO) views to enable a comprehensive examination of the whole breast tissue and increase the potential detection rate for abnormalities^11^. Nevertheless, the manual interpretation of DBT by radiologists presents inherent limitations, including the possibility of over-diagnosis and a high rate of false positives. In addition, DBT requires longer reading time than traditional 2D imaging and tends to underperform in cases of breast cancer detection in densely structured breast tissues^11,12^.

Over the years, computer-aided diagnosis (CAD) schemes have been developed to automatically detect breast abnormalities and help radiologists identify suspicious or high-risk regions within the images^13^. At the same time, the utilization of deep learning (DL) and convolutional neural network (CNN) approaches has witnessed significant growth in various computer vision applications^14^. DL models have demonstrated remarkable success in medical image tasks, including the detection of masses^15^.

CNNs have been highly successful on computer vision tasks, primarily because of their well-designed architectural structure^16^. Today, CNNs are widely used in medical imaging, including DBT for breast cancer classification. For example, Mendes et al.^17^ utilized single-slice DBT and developed a CNN model for classifying benign and malignant breast lesions. Their model was trained on 2772 augmented images and achieved an impressive testing accuracy of 93.2%. Samala et al.^18^ proposed a layered pathway evolution method for compressing a deep convolutional neural network (DCNN) in a mass classification for DBT. Through the use of transfer learning and genetic algorithms, they significantly reduced the number of neurons, parameters, and operations while still achieving a high test Area Under the Receiver Operating Curve (AUC and ROC, respectively) of 0.90. Li et al.^19^ investigated the impact of using DBT and full-field digital mammography (FFDM) with transfer learning for breast cancer mass classification. They found that transfer learning improved classification performance for both modalities.

Very recently, vision transformers (ViTs) have attracted considerable attention in computer vision. They draw their inspiration from widely-used transformers in natural language processing (NLP)^20^. ViTs address a significant drawback of CNNs, i.e., their limited ability to capture long-range dependencies and contextual information on computer vision tasks. The literature in the last few years has reported that ViTs can exceed CNN benchmarks across various tasks^16^. ViTs have also displayed their versatility in tasks other than classification, in part because they can effectively incorporate tokens that capture and preserve essential spatial information. This key attribute makes ViTs a promising direction for future applications in object detection^21^.

Unlike works on CNN, few studies have used ViTs for breast cancer detection. Ayana et al.^20^ developed a transfer learning technique based on vision transformers to classify breast mass mammograms. Their model outperformed CNN-based transfer-learning and vision transformer models trained from scratch, and achieved an estimated area under the receiver operating curve of 1 ± 0. Cantone et al.^22^ compared CNNs and ViTs, specifically Swin Transformers on mammogram classification. Although the CNNs performed better, they found that SwinV2 showed promise in terms of a reintroduced locality bias. Higher input resolution improved the Swin Transformers’ performance by making them scalable for mammography analysis. Lee et al.^23^ introduced a distinctive approach to DBT classification that differed from mammography techniques. They proposed a transformer-based deep neural network architecture combined with a convolutional neural network for breast cancer detection on DBT images that took neighboring sections into account. Their method achieved an AUC of 0.91 ± 0.03.

DL faces a multitude of challenges in DBT, including choosing between 2D and 3D models, limited computational resources for high-resolution 3D model training, the need for extensive training sets and full-resolution inputs, as well as complexities in terms of data curation and labeling, especially when attempting to capture volumetric information and accurately delineate tumor boundaries across multiple DBT slices^3^. Many machine learning tasks, particularly in medical imaging, often necessitate expert physicians to label the datasets, rendering this procedure costly and time-consuming. This limitation highlights the need to develop semi-supervised learning methods that can process a small set of labeled data and a more extensive set of unlabeled data to train models^24^.

To respond to this need, this study describes a method that uses labeled and unlabeled 2D DBT slices to detect breast tumors on 3D DBT scans. Our method employs ViTs to extract the tumor probability in each 2D slice, which is followed by post-processing that examines the relationships between consecutive slices. We compare its classification performance to a baseline model trained solely on labeled DBT slices. This paper is structured as follows: the second section presents the primary findings obtained using the different approaches, the third section discusses the results, and the last section details the methods and materials utilized.

## Results

### Dataset description

In this study, a unique dataset of DBT scans was retrospectively collected at Soroka University Medical Center, a tertiary center located in southern Israel that serves a population of 1.2 million people. The dataset comprised DBT images sourced from female patients above 18 years of age, including all breast density groups (A–D), and was acquired after local Institutional Review Board (IRB) committee approval (SOR0280-21). We excluded subjects with prior breast surgery or a tumor diagnosis, women with breast implants or pacemakers, and male subjects. In addition to the DBT images, the dataset also includes demographic information and clinical data on the imaging findings. The dataset consisted of a total of 2281 cases, including 837 positive cases (the presence of either benign or malignant tumors detected by an expert breast radiologist on the DBT scan) and 1444 negative cases with no detected lesion (Table 1). All the positive cases were confirmed by biopsy, of which 55% were malignant. Most cases comprised both CC and MLO views from the DBT scans, but a few cases only had one view. Thus, in total the dataset contained 1631 positive and 2885 negative scans. The number of slices in each scan ranged from 23 to 183, with a mean of 66 and a standard deviation of 15. The dataset consisted of scans with an inherent resolution of 70 micrometers per pixel ($\upmu$m/pixel), and each scan exhibiting dimensions of 2457 $\times$ 1996 pixels. Due to budget constraints, a breast radiologist labeled the 3D scans in a binary manner, indicating either the presence or absence of a tumor in the scan. However, the labeling did not specify the exact slices where the tumor was located within the scan. Partial labeling was carried out for approximately 10 slices in each positive scan by a breast radiologist.

**Table 1:** Summary of the dataset.

	Number of cases	Number of scans	Age
Negative	1444	2885	$55\pm 13$
Positive	837	1631	$56\pm 14$

### Classification results

The model’s performance was assessed by evaluating its results on a test set comprising 685 scans consisting of 253 positive and 432 negative scans. These scans were derived from 127 positive cases and 216 negative cases, respectively.

**Table 2:** Performance metrics of supervised transfer learning models for lesion detection in DBT.

Model (resolution)	Scan-based			Case-based			
	AUC	Sensitivity	Specificity	AUC	Sensitivity	Specificity	p-value
ResNet101 (224)	$0.784 \pm 0.037$	$0.747 \pm 0.055$	$0.666 \pm 0.044$	$0.824 \pm 0.047$	$0.801 \pm 0.068$	$0.695 \pm 0.059$	–
ViT (224)	$0.787 \pm 0.036$	$0.769 \pm 0.048$	$0.638 \pm 0.045$	$0.836 \pm 0.044$	$0.853 \pm 0.058$	$0.632 \pm 0.065$	0.576
Swin (224)	$0.835 \pm 0.033$	$0.778 \pm 0.051$	$0.766 \pm 0.039$	$0.883 \pm 0.038$	$0.838 \pm 0.062$	$0.808 \pm 0.056$	0.005
Swin (384)	$0.880 \pm 0.028$	$0.829 \pm 0.052$	$0.724 \pm 0.054$	$0.934 \pm 0.026$	$0.912 \pm 0.046$	$0.746 \pm 0.061$	$6.3\cdot 10^{-8}$
Swin (1024)	$0.878 \pm 0.030$	$0.827 \pm 0.064$	$0.794 \pm 0.053$	$0.915 \pm 0.033$	$0.864 \pm 0.056$	$0.837 \pm 0.051$	$6.6\cdot 10^{-6}$

**Figure 1:**
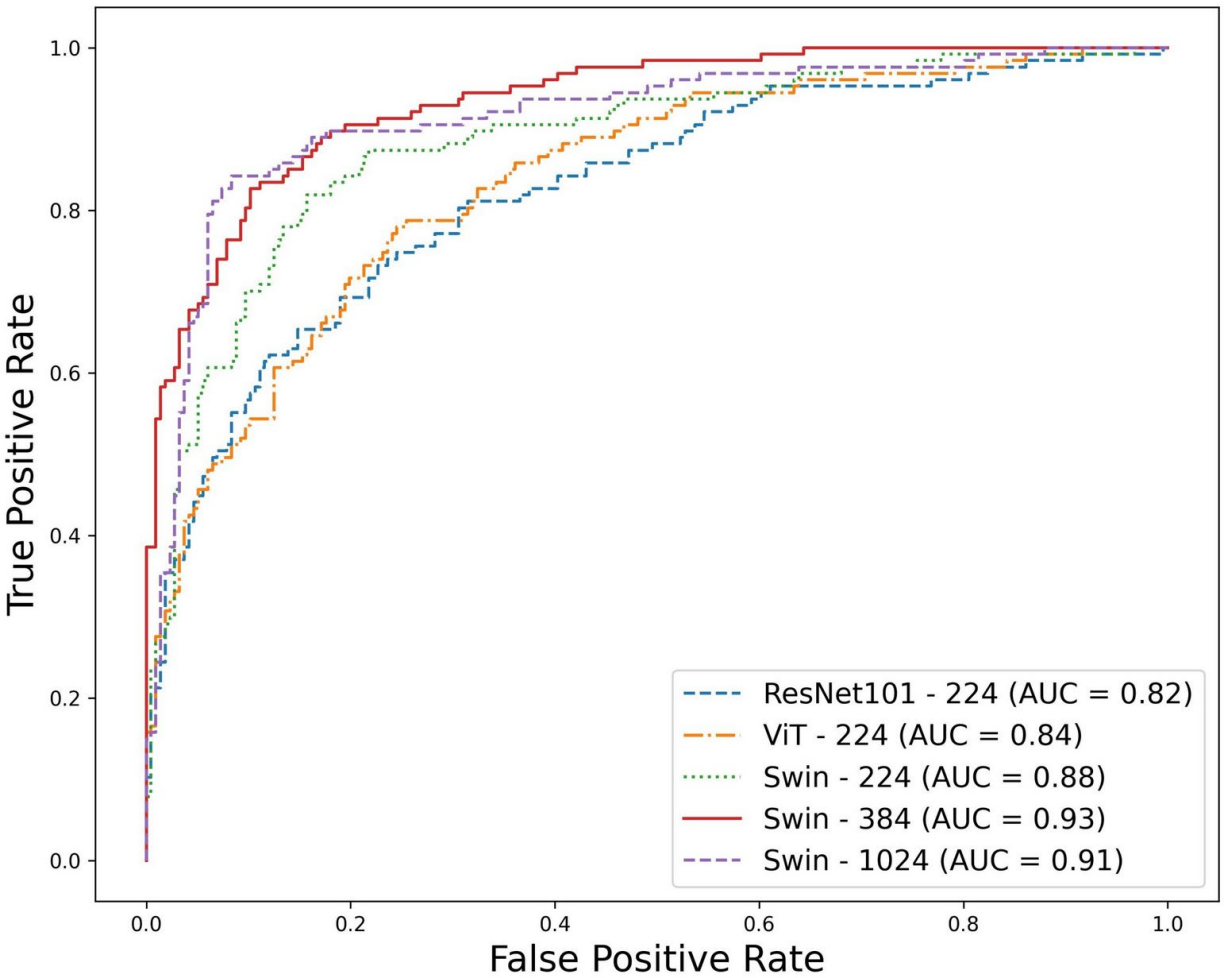
ROC curve for supervised models evaluated on the test dataset consisting of the ResNet101 with an input resolution of 224 $\times$ 224, ViT with an input resolution of 224 $\times$ 224, and the Swin Transformer with input resolutions of 224 $\times$ 224, 384 $\times$ 384, and 1024 $\times$ 1024. The AUC values for each model are indicated in the legend.

When training the model using the supervised learning approach, it became evident from the results as presented in Table 2 and Fig. 1 that the Swin transformer^25^ performed better than other architectures. Table 2 also presents the p-values of the case-based AUC metric compared to the ResNet101^26^ baseline architecture. Comparison of the ResNet101 and vanilla ViT^27^ architectures to the Swin transformer with an exact resolution of 224 $\times$ 224 indicated a statistically significant difference in AUC at p-values of 0.005 and 0.01, respectively. When the image size was increased from 224 to 384, significant improvements in AUC were observed in terms of the Swin model’s performance on the classification tasks (p-value = 0.0003), in particular when the model was initialized with weights obtained from pretraining on the ImageNet dataset. Nevertheless, when the image size was increased to 1024, no substantial enhancement in classification performance was observed, and the differences were not statistically significant. To verify the robustness of the top-performing model (Swin with 384-input resolution), we performed ten random divisions of the dataset into training, validation, and test sets, ensuring data independence across the sets. Subsequently, we calculated the average and standard deviation of the case-based AUC across all test sets, which yielded a result of 0.918 ± 0.014.

As shown in Table 3, when training the Swin Transformer with a 384-resolution model using a semi-supervised learning approach, no significant improvement was observed as compared to the supervised method.

**Table 3:** Performance metrics of semi-supervised Swin transformer with a 384 $\times$ 384 resolution in DBT lesion detection.

Model (resolution)	Scan-based			Case-based		
	AUC	Sensitivity	Specificity	AUC	Sensitivity	Specificity
Swin (384)	$0.886 \pm 0.026$	$0.874 \pm 0.048$	$0.720 \pm 0.056$	$0.932 \pm 0.025$	$0.944 \pm 0.039$	$0.744 \pm 0.056$

### Sub-group analysis

The first step in this study was to assess the model’s ability to detect lesion pathologies in scans, without distinguishing between benign and malignant lesions. Next, we analyzed the model’s performance when classifying benign and malignant lesions based on the corresponding biopsy results. Then, we investigated the model’s proficiency in detecting lesions of different sizes. Specifically, we focused on tumors measuring up to 2 cm and those exceeding 2 cm, since this threshold marks the transition between T1 and T2, the first two levels of the TNM staging system for breast cancer^28^. This distinction is important in clinical practice as it often influences the treatment approach and prognosis^29^. To ensure accuracy, the sizes of the lesions were precisely measured through ultrasound (US) imaging which was performed within 6 months after DBT. Furthermore, we analyzed the model’s performance by specifically focusing on women below and above 50 years, and by considering the inverse relationship between patient age and mammographic breast density. This relationship is crucial to explore in that increased breast density can reduce the sensitivity of mammography as an early detection tool^30^. The performances in terms of the case-based AUC are presented in Table 4. In the AUC analysis for subgroups, each positive sub-group is assessed against the negative group, offering insight into the model’s discriminative capacity across these specific clinical classifications. Both the supervised and semi-supervised Swin models had consistent, remarkable performance across a wide range of tumor types, sizes, and age groups, and exhibited no statistically significant difference in performance within these subgroups.

**Table 4:** Case-based AUC results for subgroups using Swin transformer at a 384 $\times$ 384 resolution.

		Supervised Swin	Semi-supervised Swin
Biopsy result	Malignant	$0.926 \pm 0.035$	$0.915 \pm 0.035$
	Benign	$0.942 \pm 0.034$	$0.955 \pm 0.025$
Lesion size	Diameter < 2 cm	$0.932 \pm 0.029$	$0.930 \pm 0.028$
	Diameter $\ge$ 2 cm	$0.938 \pm 0.043$	$0.940 \pm 0.038$
Age	Age < 50	$0.933 \pm 0.044$	$0.926 \pm 0.045$
	Age $\ge$ 50	$0.936 \pm 0.031$	$0.937 \pm 0.031$

### Error analysis

**Figure 2:**
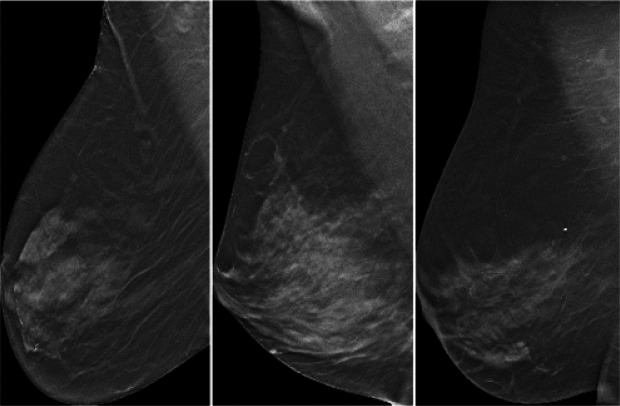
Example slices from DBT scans that were falsely identified as positive by our model. Despite being classified as an error, the images show regions of dense or heterogeneously dense breast tissue, which can often challenge automated detection systems.

This section examines the cases where our model made errors. The objective was to identify patterns in these errors for future improvement. We identified two main types of errors: false positives (FP) and false negatives (FN). To gain insights into these errors, we investigated factors such as the woman’s age, the lesion size, and the BI-RADS (Breast Imaging Reporting and Data System) score assigned by the radiologist during the initial examination. We also considered the results of the US and biopsy test conducted within six months after the interpretation of DBT. Table 5 presents the classification results based on the biopsy results, the gold standard for both methods. Using the supervised Swin method, we achieved a case-based sensitivity of 0.912. Of the positive cases, 11 out of 127 were classified as false negatives. Notably, eight of these 11 FN cases had a diameter of less than 2 cm, with an average diameter of 1.5 cm and a standard deviation of 0.9 cm. The average age of these misclassified cases was 55, with a standard deviation of 14 years. It is worth noting that the expert radiologist categorized 7 out of the 11 errors as BI-RADS 0 using the DBT scan, thus indicating the need for an additional imaging evaluation to determine the appropriate diagnosis^31^. The method employed in this study demonstrated a case-based specificity of 0.746, indicating a higher number of false positive errors. Specifically, out of the 216 negative cases, 54 were misclassified as positive. Of these 54 misclassifications, 14 cases were identified during the US examination, thus leading to subsequent biopsies. Five of these findings were confirmed as malignant in the biopsy, whereas the remaining 9 were benign. In these 14 cases, the radiologist failed to identify the findings during the DBT interpretation, whereas the model successfully detected and correctly classified the lesions. As a result, accounting for the model’s specificity based on biopsy results increased specificity. Of the remaining 40 FP errors, some cases exhibited dense breast structures and calcifications (e.g., Fig. 2). Overall, this suggests that enhancing the dataset with more negative cases with these characteristics could contribute to improving classification accuracy.

**Table 5:** Case-based detection in DBT using supervised and semi-supervised Swin transformer at 384 $\times$ 384 resolution, benchmarked against biopsy results.

Model	AUC	Sensitivity	Specificity
Supervised Swin	$0.932 \pm 0.025$	$0.910 \pm 0.048$	$0.800 \pm 0.054$
Semi-supervised Swin	$0.939 \pm 0.024$	$0.948 \pm 0.037$	$0.794 \pm 0.056$

**Figure 3:**
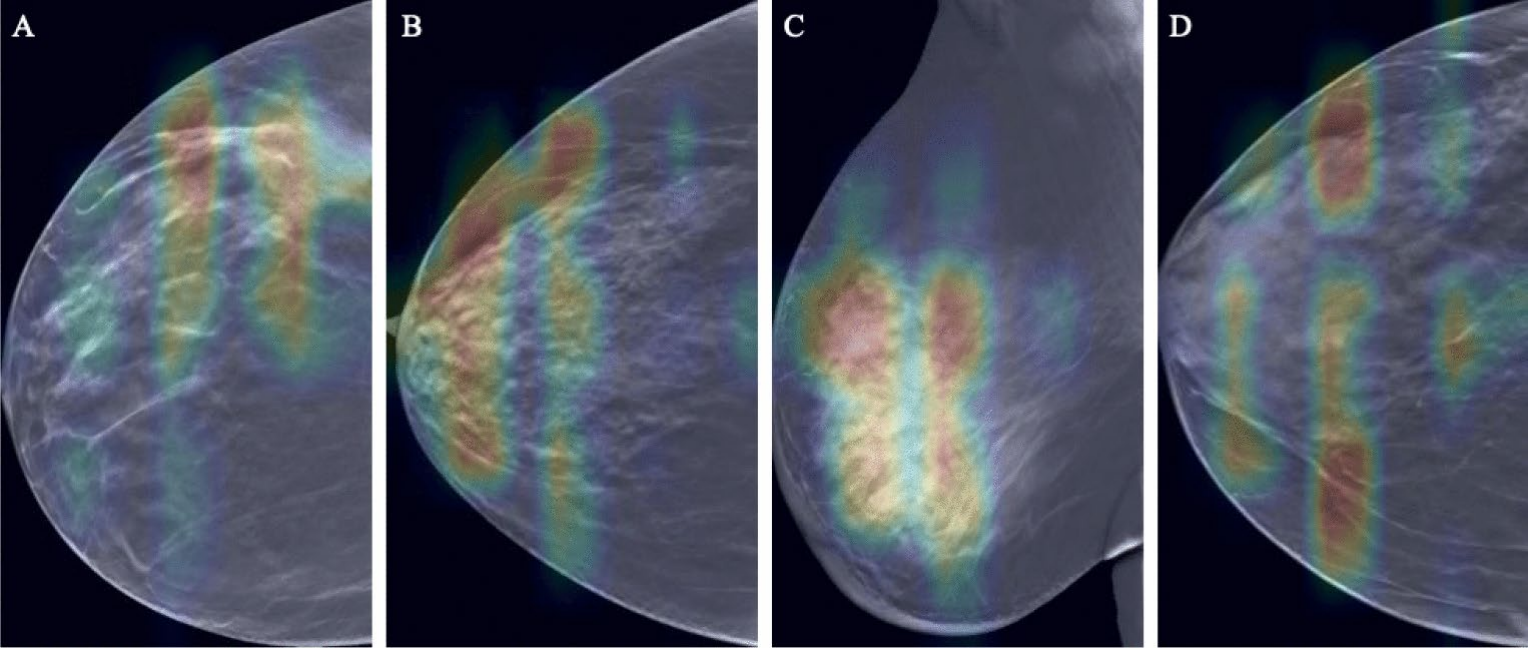
Grad-CAM visualizations of model attention in cases of misclassification. (**A**,**B**) False-positive predictions, where the model’s focus is erroneously drawn to heterogeneous or dense structures in the breast. (**C**,**D**) False-negative predictions, showing instances where the model either fails to maintain sufficient focus on the tumor or disperses attention across multiple areas.

To gain deeper insights into the model’s decision-making process during errors, we analyzed false-negative and false-positive predictions using Grad-CAM^32^ visualizations, as illustrated in Fig. 3. The heat maps revealed that in cases of false-positive errors, the model’s attention was often drawn to heterogeneous structures within the breast, which were mistakenly emphasized as potential tumors. On the other hand, false-negative errors showed that the model occasionally managed to focus on the actual tumor, but this focus resulted in borderline confidence, failing to meet the classification threshold. Alternatively, the model distributed its focus across different areas of the breast, including regions suspected of containing a tumor, but failed to focus on the specific tumor, possibly due to factors such as breast density or symmetrical abnormal structures in both breasts. Notably, the confidence levels in these errors were generally medium, further supporting the observation that the model’s attention was dispersed rather than concentrated on the tumor. By analyzing these errors through Grad-CAM, we gain valuable insights for model refinement. Understanding the conditions under which the model misclassifies enables us to explore potential improvements, such as incorporating additional training data that better represents challenging cases.

We extend the analysis to evaluate the performance of our models against biopsy results across the four BI-RADS density categories (A-D). Category A denotes breasts that are almost entirely fatty, Category B signifies scattered areas of fibroglandular density, Category C indicates heterogeneously dense breasts, and Category D represents extremely dense breasts^33^. Our test set distribution reflects these categories: Category A at 18% (62 cases), B at 38% (131), C at 33% (112), and D at 11% (38), which aligns with the population distribution in the average age group of our dataset^34^. Table 6 displays the performance metrics for the supervised model across these breast density groups. Literature suggests that DBT is particularly effective in detecting breast cancer in dense breast tissues due to its three-dimensional imaging capabilities^35^, which are consistent with our findings. The error rate exhibited by our model remained steady across all breast density categories. There was a slight decline in sensitivity when evaluating denser breast tissue, which was accompanied by an incremental rise in specificity.

**Table 6:** Performance metrics of the supervised Swin model by BI-RADS density categories against biopsy results.

BI-RADS density	Error rate	Sensitivity	Specificity
A	12.9% (8)	$0.944 \pm 0.056$	$0.788 \pm 0.129$
B	16.8% (22)	$0.925 \pm 0.058$	$0.751 \pm 0.101$
C	16.1% (18)	$0.832 \pm 0.105$	$0.835 \pm 0.085$
D	13.2% (5)	$0.875 \pm 0.125$	$0.821 \pm 0.141$

## Discussion

Digital breast tomosynthesis is an innovative imaging modality that significantly improves traditional 2D mammography. DBT provides a three-dimensional view of the breast, thus allowing for enhanced lesion visibility, reduced tissue overlap, and improved diagnostic accuracy. As a result, DBT has demonstrated superior sensitivity and specificity in detecting breast cancers, particularly in women with dense breast tissue. Its ability to detect otherwise occult cancers has proven to be a transformative advance in early diagnosis and treatment, that can potentially lead to better patient outcomes and reduced mortality rates. The integration of CAD schemes holds the potential to augment the efficacy of DBT in breast cancer detection. By harnessing the power of deep learning algorithms, CAD systems can aid radiologists in identifying and characterizing suspicious lesions, thus reducing the likelihood of oversight and misdiagnosis. These intelligent systems serve as invaluable second opinions^36^ by providing radiologists with an additional layer of analysis that can contribute to identifying subtle abnormalities that might be missed during manual interpretation. Thus, the integration of CAD systems with DBT can decrease radiologist workload while improving diagnostic accuracy and efficiency significantly.

In this work, we proposed a novel CAD scheme for analyzing and classifying DBT scans. The novelty of the proposed CAD scheme lies in applying the Swin Transformer to DBT image analysis. Unlike traditional CNNs, vision transformers capture both local and global contexts through a unique attention mechanism, enabling efficient processing of large images and 3D data with high accuracy. The Swin Transformer’s ability to handle varying resolutions and extract hierarchical features leads to superior classification performance, particularly in detecting subtle differences critical for accurate DBT diagnosis. These innovations set a new benchmark for CAD systems in breast cancer detection, marking a clear advancement over existing methods. In addition, this method offers significant potential to enhance radiological practice in breast cancer detection via DBT. By improving diagnostic accuracy and reducing false positives and negatives, the system can ease the diagnostic burden on radiologists and contribute to more efficient workflows. Furthermore, the system can serve as a second reader, offering an additional layer of validation that complements radiologists’ evaluations and further mitigates diagnostic errors. The integration of attention mechanisms not only enhances interpretability but also builds trust by allowing radiologists to see the model’s focus areas. This transparency is crucial for AI adoption in clinical settings, where the system’s ability to standardize diagnostics and reduce inter-observer variability can ultimately improve patient outcomes.

**Figure 4:**
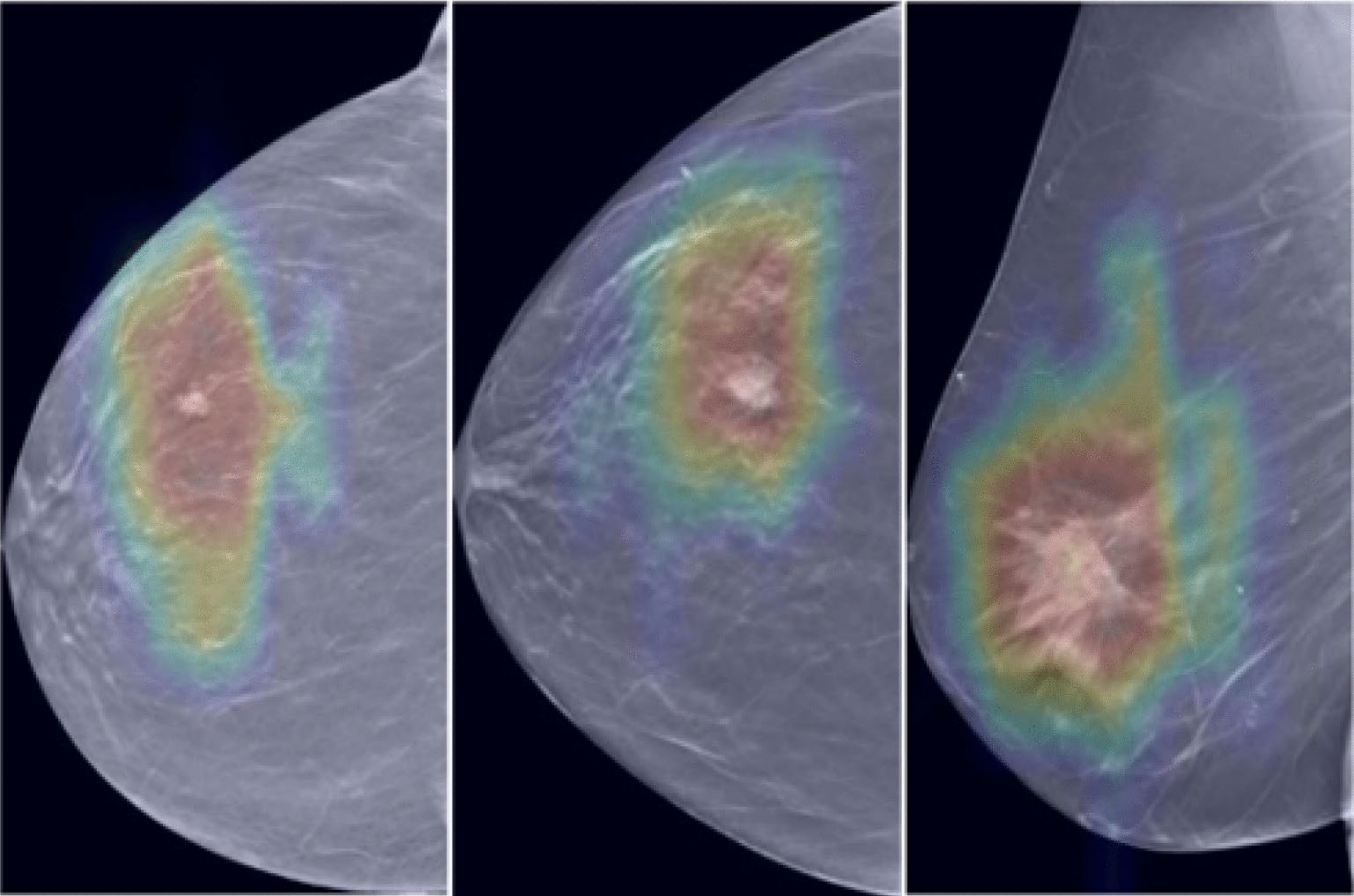
Visualization examples of the Grad-CAM heat maps generated from the last attention layer of the Swin Transformer. These maps highlight the salient regions within the DBT images that the model prioritizes during its inference process, thus demonstrating the model’s attention mechanism and its capacity to pinpoint areas of potential clinical significance.

The method was trained on a DBT dataset comprising 3,831 scans and was evaluated on a separate test set of 685 DBT scans. We trained a classification model using supervised learning in the first phase and compared different architectures. The experimental results clearly showed that the Swin architecture outperformed the other architectures. Swin demonstrated significantly higher AUC scores than the ResNet101 and vanilla ViT architectures at a 224 $\times$ 224 resolution (p < 0.01), and when increasing the image size to 384 improved Swin’s AUC (p = 0.0003), particularly when initialized with ImageNet weights. The Swin architecture achieved the highest case-based AUC score of 0.934 ± 0.026 at a 384 resolution and demonstrated a remarkable improvement compared to ViT. The better performance of the Swin Transformer can be attributed to several key factors. The Swin Transformer’s window-based approach and shifted windowing scheme enable efficient local attention within each window while capturing crucial global information. This allows the model to process large-scale images and maintain contextual understanding effectively. In addition, the patch merging process in the Swin Transformer strikes a balance between detailed information and overall context by intelligently consolidating neighboring patches. This consolidation enhances the model’s ability to capture fine-grained and high-level features, as observed in DBT applications. The integration of attention mechanisms holds promising potential to enhance DBT applications by effectively identifying and highlighting regions of interest, thereby significantly increasing the explainability and accuracy of diagnoses in the medical domain. Attention heat maps can be generated using the Grad-CAM algorithm^32^, as shown in Fig. 4. These heat maps visually represent the salient regions within the DBT images that the layer prioritizes during its inference process. The heatmaps derived from the model’s final attention layer yield significant insights, playing essential roles in clinical settings. They enhance interpretability and clinician trust by visually representing the decision-making process, pinpointing DBT scan regions critical for classification, and thereby facilitating its use as a diagnostic tool. Additionally, these heatmaps highlight suspicious areas, guiding radiologists to potentially problematic regions, which can streamline the review process and improve diagnostic accuracy. Moreover, they act as a qualitative tool for validating the model’s precision, allowing clinicians to compare highlighted regions against actual tumor locations to assess the model’s detection capabilities. However, integrating such visualization tools into clinical practice demands thorough evaluation, including addressing challenges, securing approvals, and training clinicians for accurate interpretation. These steps are vital to effectively leveraging the attention mechanism visualizations for cancer diagnosis enhancement.

Increasing the input resolution from 224 to 384 in the Swin architecture improved classification performance. The higher resolution resulted in less degradation of information within the scan than lower resolutions, thereby preserving subtle features such as small tumors. However, there was no significant difference in classification performance when the resolution was increased to 1024. Training the model at this higher resolution while utilizing an initialization based on a model pre-trained on a lower resolution (384) led to a marginal decrement in performance. This outcome can be attributed to the small amount available data and the growing risk of overfitting associated with larger resolutions. In general, it is advisable to minimize the reduction in input resolution as much as possible when working with DBT scans. This approach helps to mitigate damage to tumor and tissue features, since higher resolutions demand substantial memory and significant computing power.

In the second phase of the study, we employed the semi-supervised Mean Teacher method for training to enhance model performance. This did not significantly improve the DBT results compared to the supervised method. The model’s performance did not substantially improve despite incorporating the unlabeled data into the learning process. This outcome suggests that additional unlabeled data might not provide the expected boost in accuracy or generalization capabilities for this digital breast tomosynthesis classification task. Further investigation and experimentation are warranted to explore alternative approaches or different semi-supervised algorithms to achieve better performance in this context.

The model’s performance was evaluated on different subgroups in terms of tumor type (benign or malignant), tumor size, and subject age. However, the analysis did not reveal any statistically significant differences in performance. Surprisingly, even with smaller tumors that covered a smaller area in the input image, lowering the resolution did not adversely affect the model’s detection ability. Similarly, despite the higher breast density and challenges associated with mammography interpretation in women over 50, the model exhibited comparable classification performance across age groups. To further enhance the model’s performance, enriching the dataset with challenging cases that pose difficulties for expert radiologists could improve overall performance and address potential misidentification within specific subgroups.

This work has a number of limitations. The dataset was collected from a single medical center, and all the scans were acquired from a machine manufactured by a single vendor (Hologic). Our approach involved binary classification without distinguishing between benign and malignant tumor types. However, we examined this factor and found that the model successfully identified both tumor types without any significant statistical difference. Because the temporal analysis focused on the probability of tumor presence within each two-dimensional slice comprising the three-dimensional scan, we did not explore the spatial interactions among features within the scan, which could have potentially improved classification performance. In our initial endeavor, we attempted to construct a 3D CNN from scratch. However, a lack of data compromised the model’s efficacy, inadequate optimization due to limited computational resources, and the challenges posed by the high-resolution 2D inputs and the varying depth of the 3D dimension. The prevailing literature indicates that while DBT occupies a quasi-3D space, most studies have favored 2D models that treat DBT slices as independent images. Ideally, a 3D model that processes this volumetric data more effectively captures inter-slice features. However, the high-resolution nature of DBT scans requires significant computational resources, making such models impractical for general use. Additionally, given that 3D CNNs depend on reconstructed images, it is still uncertain if they will consistently surpass 2D CNNs, with inconclusive results to date^3^. When benchmarked against the approach by Lee et al.^23^, which examines features from adjacent slices, our model exhibited enhanced performance. This underscores the Swin Transformer’s effectiveness in identifying both local and global spatial features essential for tumor detection, thus validating its suitability for classifying DBT scans.

In future work, we aim to investigate the use of semi-supervised techniques for tumor classification in DBT. Specifically, we intend to explore the impact of varying ratios of labeled and unlabeled data on classification performance. We will also enhance the existing dataset by employing both conventional and artificial methods. These extensions will contribute to a more comprehensive understanding of the potential of semi-supervised methods for tumor classification within the context of DBT.

## Methods

The methodology involves data pre-processing, wherein 3D DBT scans are converted into 2D images and normalized. This is followed by implementing supervised and semi-supervised learning techniques using state-of-the-art Vision Transformer architectures and ResNet101 for feature extraction and estimating tumor presence probabilities in each 2D slice of the DBT scan. Finally, post-processing and temporal analysis are conducted on the model predictions to generate tumor detection scores for the entire scan.

### Data pre-processing

The current study involved preprocessing the Digital Imaging and Communications in Medicine (DICOM) files for analysis. Initially, the 3D scans were separated into 2D 16-bit PNG images to reduce the model’s computational complexity. Then, we employed fixed thresholding for breast boundary segmentation. To reduce background noise, we cropped the images, while aiming for optimal content preservation. The images were then resized to match the resolution requirements of the architectures. To standardize the pixel values, we normalized by using the mean and standard deviation of each channel. To exploit the pre-trained networks trained on natural color images, we replicated the first channel three times, mimicking a three-channel color image. This allowed us to harness the benefits of the pre-trained networks effectively. The dataset was divided randomly into training, validation, and test sets at 70%, 15%, and 15%, respectively, following standard practices in machine learning experiments. Special attention was paid to ensuring that all scans of the same subject remained within a single dataset to maintain data independence.

### Model architecture

We employed two state-of-the-art vision transformers models pre-trained on the ImageNet-21K dataset^37^ as feature extractors: vanilla ViT^27^ and the Swin transformer^25^. ViT has revolutionized image recognition by combining the transformer’s self-attention and patch-based tokenization to capture global image information and long-range model dependencies. The Swin transformer improves locality through nonoverlapping window-based self-attention, thus facilitating hierarchical representations and window-to-window communication in subsequent layers^20^. The ResNet101 architecture was selected as a benchmark feature extractor given its cutting-edge ranking among CNN architectures^26^. The output of the feature extractor network was then fed into a fully connected (FC) layer comprising two neurons with sigmoid activation, hence enabling the final classification task of distinguishing between negative and positive cases. The networks were provided with a DBT slice image as input, and the resulting output was a tumor detection score obtained by utilizing the sigmoid function. To account for the temporal information between the two-dimensional slices, and derive inferences from a complete DBT scan, we implemented a post-processing model. A detailed description of this model is provided in the inference subsection.

### Training process

After partitioning the data into subsets, augmentations were applied to the training dataset, including color augmentation, flipping, and rotation to enhance the size and diversity of the training data.

For all the architectures we applied transfer learning from ImageNet-21K, an established technique for training DL networks with limited datasets that has demonstrated remarkable effectiveness^38^. However, we also used network retraining due to disparities between our DBT and the original ImageNet datasets. The pre-trained weights, acquired with input resolutions of either 224 $\times$ 224 or 384 $\times$ 384 pixels, underwent fine-tuning by loading them and enabling all layers to learn, thereby adapting the networks to our specific task.

**Supervised:** In the initial step, we conducted supervised training on the labeled images to predict tumor findings in the DBT scan. Only labeled two-dimensional slices were utilized for training the model, i.e., approximately 10 slices from each scan. The training phase thus consisted of 13,275 positive and 20,260 negative images belonging to 1147 positive and 2026 negative scans, respectively. To optimize the performance of the model, we constructed a hyperparameter tuning validation set. During training, we used a batch size of 32, which allowed for efficient computation and effective gradient updates. The initial learning rate was set to $10^{-4}$. We applied a cosine scheduler with a warm-up to adjust the learning rate throughout the training process dynamically. We utilized the AdamW optimizer for optimization, which has shown to be effective in training DL models^39^. The loss function was binary cross-entropy, which is well-suited for binary classification tasks. We employed a weighted loss function to address the challenge of imbalanced data. This approach assigns higher weights to the minority class samples, thereby providing a better balance during training and reducing the bias towards the majority class. Throughout the training process, we employed early stopping with a patience of 5 epochs, which monitors the validation performance and terminates training when no further improvement is observed.

**Figure 5:**
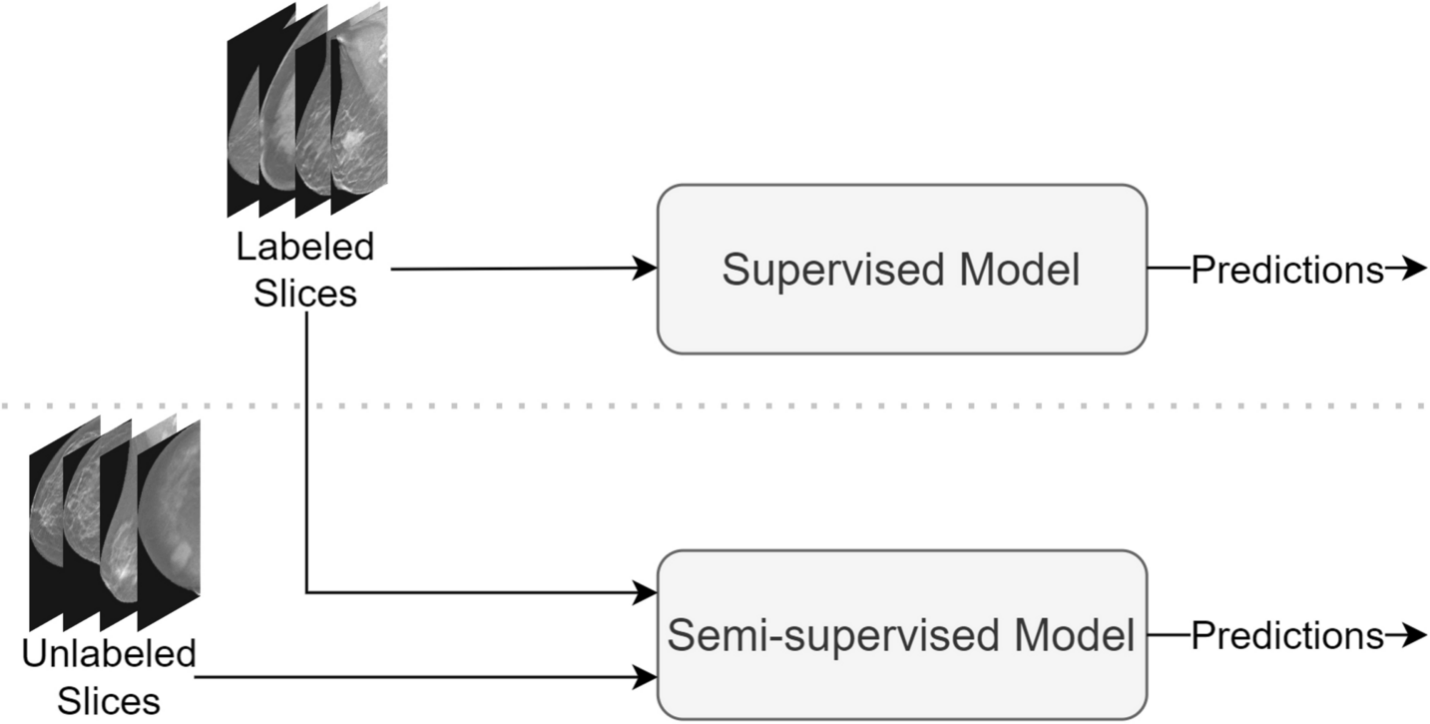
A block diagram of the approach for model training. The upper part of the figure represents stage 1: Supervised training on labeled data followed by model prediction. The lower part shows stage 2: Semi-supervised training employing the mean teacher algorithm that leveraged both labeled and unlabeled data, and the subsequent model prediction.

**Semi-Supervised:** Considering the substantial labeling costs associated with three-dimensional DBT scans, a partial labeling strategy was employed. In the training set, approximately 10 slices per scan were selected for labeling, resulting in a subset of unlabeled slices. Specifically, 68,280 2D slices were obtained from the positive scans that remained unlabeled. We opted to utilize a semi-supervised learning method to address this challenge when training our model. This approach enables the incorporation of labeled and unlabeled data during training, thereby capitalizing on the available information and optimizing model performance. The Mean Teacher algorithm is a powerful approach to semi-supervised learning that can handle large amounts of unlabeled data to improve the performance of deep learning models. Mean Teacher accelerates learning and enhances classification accuracy by averaging model weights to create a target-generating teacher model. This algorithm has been extensively evaluated was shown to be superior to other approaches in terms of speed and performance^40^. We employed the Mean Teacher method on the Swin Transformer architecture with an input size of 384 to train our models (Fig. 5). This involved parallel training of the model with labeled and unlabeled data, using two different loss functions. We utilized the binary cross-entropy loss, which is often employed for classification tasks, to optimize the model’s predictions for the labeled data. This loss function compared the model’s outputs to the ground truth labels of the labeled samples. We encouraged accurate classification of the labeled data by minimizing the binary cross-entropy loss. Simultaneously, we incorporated a consistency cost for the labeled and unlabeled data. The consistency cost was calculated as the mean squared error (MSE) between the predictions of the student model and the predictions of the teacher model. The teacher model’s predictions were obtained by applying an exponential moving average (EMA) to the student model’s predictions. This consistency cost helped encourage smooth and consistent predictions across different augmentations of the same input, thus providing regularization and improved generalization. We introduced noise during each training batch to enhance the model’s robustness. This noise was generated by applying augmentations such as flipping, rotations, and color. By introducing variations in the input data, we aimed to make the model more resilient to noise and improve its ability to generalize to unseen samples. We optimized the consistency cost weight to 4 and the EMA decay to 0.991 using the validation set to fine-tune the tradeoff between the classification and consistency costs. This weight determined the relative importance of the two loss functions and was crucial to achieving optimal performance on the labeled and unlabeled data. The hyperparameter tuning process aligned with the methodology for training the supervised model using the validation set. To address the imbalanced data, we implemented a strategy that focused on achieving class equality within each mini batch.

### Inference

**Figure 6:**
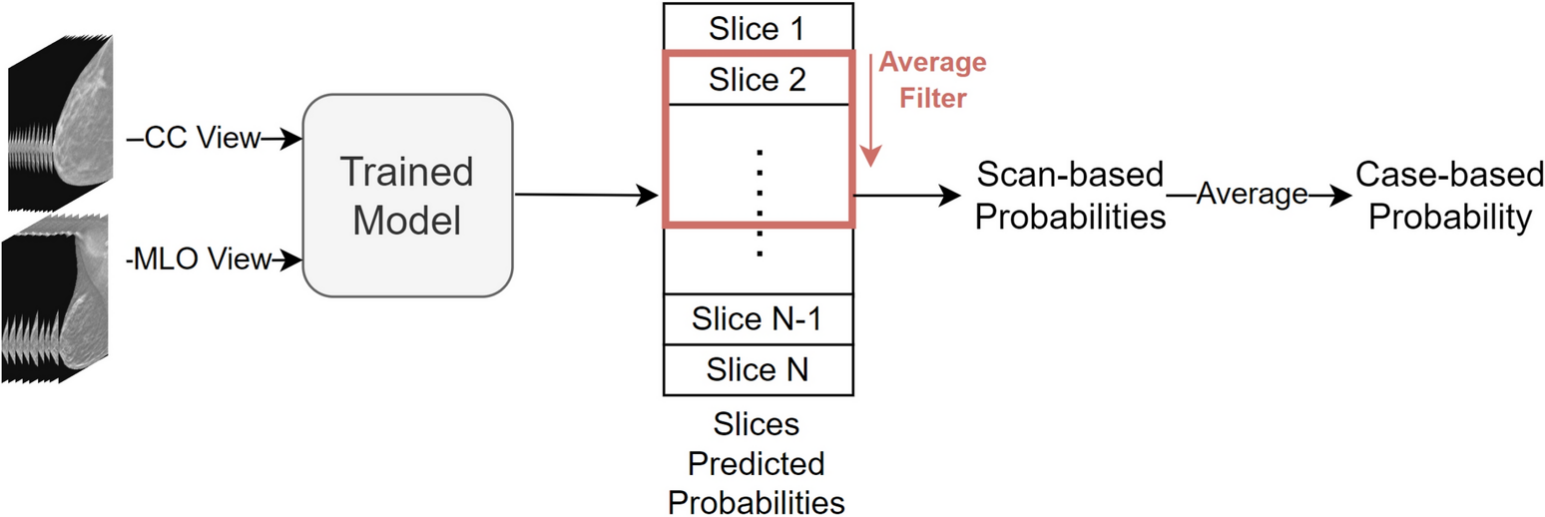
Schematic of post-processing and temporal analysis for DBT scans. Each slice is scored for tumor likelihood, with a temporal analysis that applied a moving average of 8 across adjacent images. The peak average denotes the scan score. The case score is the average of both CC and MLO scan scores.

During model testing, the 3D scans were divided into two-dimensional slices to extract features and predict probabilities using the trained model. Subsequently, the probabilities of all slices within the same scan underwent post-processing and temporal analysis (Fig. 6) to obtain a prediction for tumor findings across the entire scan. The classification results at the scan-based level were derived from this outcome. At the case-based level that comprised two scans (CC and MLO) per case, the average of the scores from both scans within the same case represented the case’s probability. This yielded the classification results at the case-based level.

### Post-processing and temporal analysis

Each slice of the DBT scan yielded a score ranging from 0 to 1 from the trained model output, indicating the likelihood of tumor presence. The probabilities of the scan were then subjected to temporal analysis. In this stage, our objective was to classify the complete scan that comprised a variable number of two-dimensional images. To do so, we applied a moving average of 8 to the probabilities of all eight adjacent images within the scan. The highest average score obtained from the filter represented the probability score of the scan. We fine-tuned all parameters, including the decision threshold, to ensure optimal performance through optimization using a separate validation set.

### Implementation details

Our deep-learning models were implemented on a Linux server with an Nvidia A40 GPU using Python programming, with the PyTorch framework for neural network development and the Hugging Face transformers package. The TensorFlow library was used for ResNet101 training.

### Performance metrics and statistical analyses

The model’s performance was evaluated using quantitative performance metrics and statistical measures commonly employed in machine learning, i.e., the AUC, sensitivity, and specificity, all of which were computed at the 95% confidence interval. The confidence intervals for sensitivity and specificity were calculated using the bootstrap method. The DeLong test^41^ was employed to compare the AUCs of different algorithms and calculate the confidence intervals for the AUC results.

## Data Availability

This paper summarizes the unique contributions introduced in the current study. For additional clarifications or inquiries regarding further details, communication may be directed to the corresponding author upon reasonable request. The datasets collected and analyzed in this study were obtained with the approval of the local IRB committee in collaboration with Soroka Medical Center, Beer-Sheva, Israel. These datasets are not publicly accessible, in accordance with ethical guidelines and constraints, to safeguard privacy.
